# Short-segment fixation and transpedicular bone grafting for the treatment of thoracolumbar spine fracture

**DOI:** 10.3389/fsurg.2022.1039100

**Published:** 2023-01-11

**Authors:** Zhi-Wen Luo, Wei-Jie Liao, Bo-Lin Sun, Jia-Bao Wu, Ning Zhang, Yu Zhang, Shan-Hu Huang, Zhi-Li Liu, Zhi-Hong Zhang, Jia-Ming Liu

**Affiliations:** ^1^ Department of Orthopaedic Surgery, The First Affiliated Hospital of Nanchang University, Nanchang, China; ^2^ Institute of Spine and Spinal Cord, Nanchang University, Nanchang, China; ^3^ Department of Radiology, The First Affiliated Hospital of Nanchang University, Nanchang, China

**Keywords:** thoracolumbar fracture, transpedicular bone grafting, single-Segment, surgery, pedicle screw

## Abstract

**Purpose:**

Thoracolumbar fracture is one of the most common fractures of spine. And short-segment posterior fixation including the fractured vertebra (SSPFI) is usually used for the surgical treatment of it. However, the outcomes of SSPFI for different types of thoracolumbar fractures are not clear, and whether it is necessary to perform transpedicular bone grafting is still controversial. This study was conducted to determine the clinical efficacy of SSPFI for the treatment of different types of single-level thoracolumbar fracture, and make clear what kind of fractures need transpedicular bone grafting during the surgery.

**Methods:**

Patients with single-level thoracolumbar fracture undergoing SSPFI surgery between January 2013 and June 2020 were included in this study. The operative duration, intraoperative blood loss, anterior vertebral height ratio (AVHR) and anterior vertebral height compressive ratio (AVHC) of the fractured vertebra, local kyphotic Cobb angle (LKA), vertebral wedge angle (VWA) and correction loss during follow up period were recorded. Outcomes between unilateral and bilateral pedicle screw fixation for fractured vertebra, between SSPFI with and without transpedicular bone grafting (TBG), and among different compressive degrees of fractured vertebrae were compared, respectively.

**Results:**

A total of 161 patients were included in this study. All the patients were followed up, and the mean follow-upped duration was 25.2 ± 3.1 months (6–52 months). At the final follow-up, the AVHR was greater, and the LKA and VWA were smaller in patients with bilateral fixation (6-screw fixation) than those with unilateral fixation (5-screw fixation) of AO type A3/A4 fractures (*P *< 0.001). The correction loss of AVHR, LKA and VWA in fractured vertebra were significantly great when preoperative AVHC was >50% (*P *< 0.05). For patients with AVHC >50%, the correction loss in patients with TBG were less than those without TBG at the final follow-up (*P *< 0.05).

**Conclusions:**

SSPFI using bilateral fixation was more effective than unilateral fixation in maintaining the fractured vertebral height for AO type A3/A4 fractures. For patients with AVHC >50%, the loss of correction was more obvious and it can be decreased by transpedicular bone grafting.

## Introduction

Thoracolumbar fractures are the most common type of fractures of the spine and mostly caused by high-energy injuries (1), such as motor vehicle accidents and falls. Such fractures may cause paralysis, pain, deformity and loss of function (2). Short-segment posterior fixation (SSPF) is a commonly used surgical method for the treatment of thoracolumbar fractures (3, 4). Traditional SSPF includes two proximal pedicle screws and two distal pedicle screws fixation, and is associated with a high rate of implant failure (5). To provide more support for the anterior column and increase the stability of the spinal column (6), short-segment posterior fixation including the fractured vertebra (SSPFI) has been used as an effective alternative, with biomechanics advantages over the traditional SSPF (7). El Khateeb et al. (8) reported that short-segment fixation with screws at the fracture level had similar clinical and radiological outcomes comparing with long-segment fixation in the treatment of thoracolumbar fractures. Basaran et al. (9) conducted a finite element analysis to compare short-segment fixation with long-segment fixation for the treatment of thoracolumbar fracture, and found that short-segment fixation was sufficient to stabilize fractures at the thoracolumbar junction. Norton et al. (7) performed a study to determine the biomechanical differences between four and six-screw short-segment constructs for the management of an unstable L1 fracture, and the results indicated that six-screw construct with screws in the fractured vertebra was more rigid than a four-screw construct.

However, for patients with single-level thoracolumbar fractures, excessive compression of the fractured vertebra preoperatively is linked to increasing risk of correction loss after SSPFI (10). It also remains controversial whether it is necessary to perform transpedicular intracorporeal bone grafting (TBG) during the surgery of SSPFI (11, 12). Hence, a retrospective study was performed to evaluate the effectiveness of SSPFI in the treatment of single-level thoracolumbar fracture with unilateral and bilateral fixation of fractured vertebra, and to compare the results of SSPEI among different compression degrees of the fractured vertebra. Additionally, the efficacy of TBG for the treatment of thoracolumbar fracture was determined in this study.

## Materials and methods

Patients with single-level thoracolumbar fractures who underwent SSPFI surgery between January 2013 and June 2020 were included in this study. This study was approved by the ethics committee of first affiliated hospital of Nanchang university and all patients provided informed consent before participation.

All patients underwent surgery within 2 weeks after injury (1–14 days, average: 3.8 days). The thoracolumbar AOSpine injury score (TL AOSIS) (13) was used for surgical decision making. Patients with a TL AOSIS of three or less were given with conservative treatment, and patients with a TL AOSIS of more than five received surgical intervention. Operative or non-operative treatment was acceptable for injuries with a TL AOSIS of four or five (14). The inclusion criteria were: (1) single-level thoracolumbar fractures; (2) fresh fractures within 2 weeks; (3) underwent short-segment posterior fixation including the fractured vertebra. Patients who were diagnosed with severe osteoporosis, pathological fracture caused by infection and tumor were excluded from this study.

According to the AO spine classification (15), there were 79 patients with type A3 fracture and 82 with type A4 fracture. In terms of the fracture levels, 37, 44, 46 and 34 patients were at the level of T11, T12, L1 and L2, respectively. According to the TL AOSIS, there were 64 patients with a TL AOSIS of six, 56 patients with a TL AOSIS of seven and 41 patients with a TL AOSIS of nine.

### Preoperative measurement

All patients underwent x-ray examination, computed tomography scan (CT) and magnetic resonance imaging (MRI) of the thoracolumbar spine prior to surgery. The preoperative local kyphotic Cobb angle (LKA), vertebral wedge angle (VWA) and the anterior vertebral height (AVH) were measured. In addition, the anterior vertebral height ratio (AVHR) and the anterior vertebral height compressive ratio (AVHC) of the fractured vertebra were calculated.

**Figure 1:**
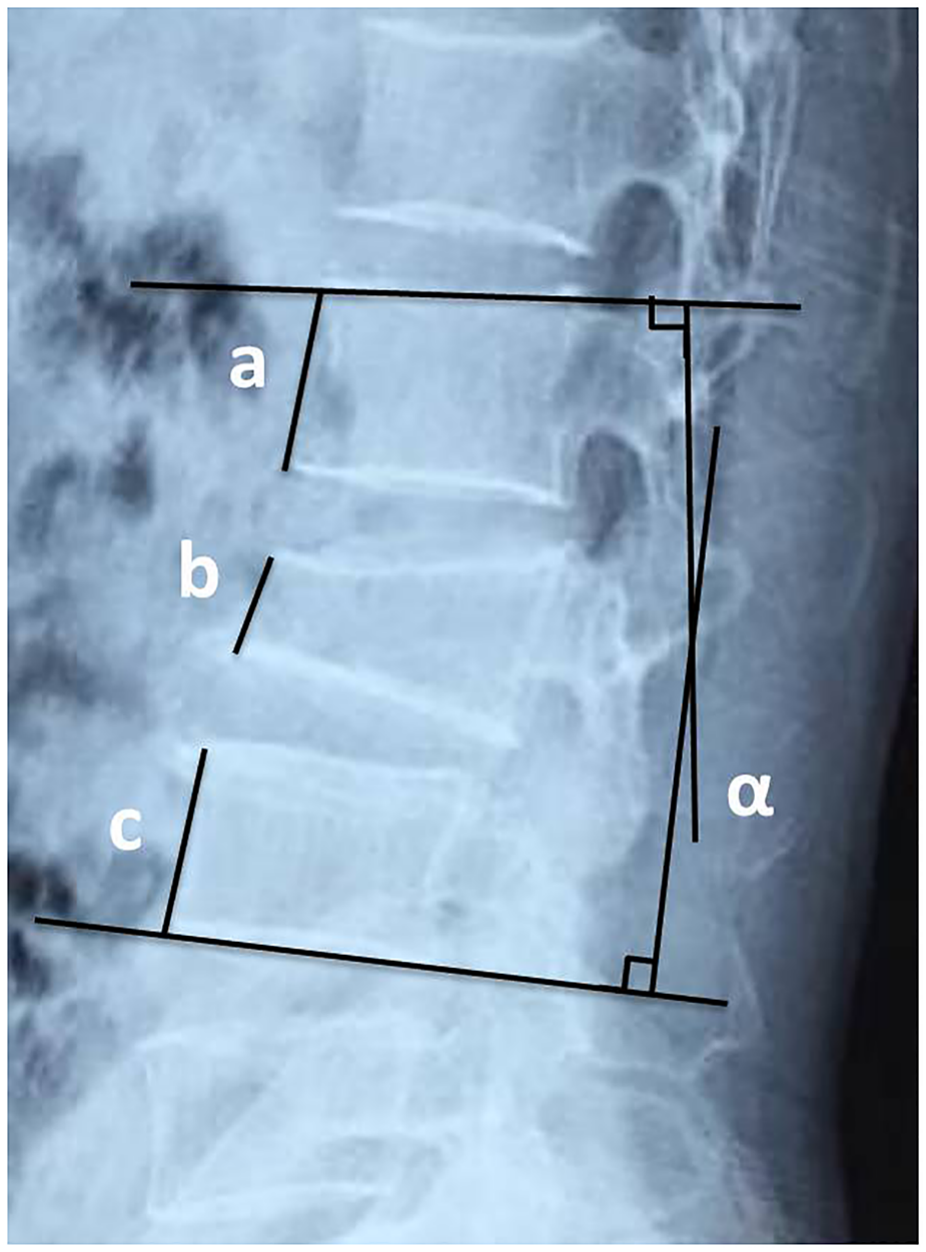
The schematic diagram of AVHR and AVHC. AVHR = 2b/(a + c), AVHC = 1-AVHR. Angle *α* represents the local kyphotic Cobb angle (LKA) and angle *β* is the vertebral wedge angle (VWA). AVHR, the anterior vertebral height ratio of the fractured vertebra. AVHC, the anterior vertebral height compressive ratio of the fractured vertebra.

The LKA was measured between the proximal end plate of the vertebra above the fractured vertebra and the distal end plate of the vertebra below the fractured vertebra. The VWA was measured between the proximal and distal end plate of the fractured vertebra. The AVHR was defined as the AVH of the injured vertebra in comparison with the mean AVH one above and one below the fractured vertebra. AVHC equals one minus AVHR (Figure 1).

### Surgical procedure

All patients were under general anesthesia in the prone position. A posterior midline incision was performed. The injured vertebra and two adjacent vertebrae were exposed. A pair of pedicle screws were inserted into the upper and lower normal vertebral bodies, respectively. Bilateral rods were properly braced. The pedicle screws were implanted on bilateral sides of the pedicles or unilateral side due to surgeon's personal preferences/ pedicle fracture. Allograft combined with autograft harvested from decompression were used as graft bone. If patient with AVHC >50%, transpedicular bone grafting was performed on fractured vertebral body bilaterally before pedicle screw insertion.

For patients with thoracolumbar burst fracture complicated with neurological deficits, posterior unilateral fenestration decompression was conducted to alleviate the dural sac compression. Part of unilateral lamina and medial inferior facet of the superior vertebra were resected, and the posterior wall of the fractured vertebral body was tamped ventrally for reduction. After that, the rods were reinstalled and temporarily tightened after proper distraction reduction performing. Finally, all screws were locked following satisfactory reduction of the fractured vertebra determined by C-arm (the anterior height of fractured vertebra restored to 90% of adjacent vertebral height). A drainage tube was placed in the surgical site and the incision was sutured layer by layer.

### Postoperative management

The drainage tube was removed 24–48 h after surgery, depending on the drainage volume. Postoperative x-ray examination of the thoracolumbar spine was regularly performed during the follow-up at 1, 3, 6 and 12 months. The operative duration, intraoperative blood loss and postoperative complications were recorded. The preoperative, postoperative and final follow-upped AVH, VWA and the LKA were measured. The thoracolumbar AOSpine injury score (TL AOSIS) and visual analog scale (VAS) score were evaluated for all patients.

According to the different strategies of pedicle screw placement in fractured vertebra, patients were divided into unilateral fixation and bilateral fixation groups. And the surgical results between these two groups were compared. Additionally, patients were divided into three groups based on the preoperative AVHC of the fractured vertebra: group A, AVHC <30%; group B, AVHC ranged from 30% to 50% and group C, AVHC >50%. The correction loss of AVHR, LKA and VWA at the last follow-up was compared among these groups. Finally, for patients undergoing bilateral fixation, according to whether the transpedicular bong grafting (TBG) was performed, they were divided into SSPFI group and SSPFI plus TBG group. And the postoperative outcomes between these two groups were compared.

### Statistical analysis

The SPSS 23.0 software package (IBM Corp., Armonk, NY, USA) was used for the data analysis. The preoperative and postoperative AVHR, VWA and LKA were compared using the paired data *t-test*. The *t-test* was used to compare the operative duration, intraoperative blood loss, AVHR, LKA, VWA and correction loss between groups. The correction loss of AVHR, VWA and LKA were compared through one-way *ANOVA* analysis of variance and the least significant difference *t-test* between three groups. A value of *P < *0.05 denoted statistical significance in all tests.

## Results

A total of 161 patients with single-level thoracolumbar fractures who underwent SSPFI were included in this study. There were 92 males and 69 females, aged 20–59 years (average: 48.5 years). The operative duration was 65–305 min (mean: 130.5 ± 40.3 min) and the intraoperative blood loss was 50–600 ml (mean: 221.4 ± 81.3 ml). All the patients were followed up, and the mean follow-up duration was 25.2 ± 3.1 months (6–52 months). Postoperative wound infection occurred in 7 patients (4.35%), and resolved after wound dressing and antibiotics treatment. The VAS score significantly decreased postoperatively.

### Unilateral vs. bilateral fixation for fractured vertebra

**Figure 2:**
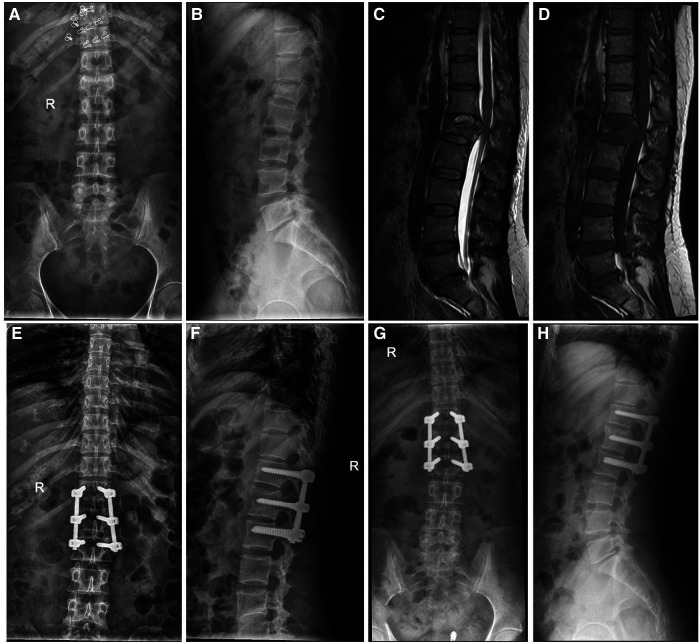
A 40-year-old female patient with L1 fracture underwent short-segment fixation with bilateral pedicle screw fixation in the fractured vertebra. (**A–D**) The preoperative x-ray and MRI images showed burst fracture of the L1 vertebra. The compression degree of the injured vertebra was 40%. (**E,F**) x-ray examination, conducted immediately after surgery, showed that the height of the L1 vertebra was recovered and the kyphosis was corrected. (**G,H**) Postoperatively (1.5 years), x-ray examination showed that the height of the L1 vertebral body was well maintained.

**Figure 3:**
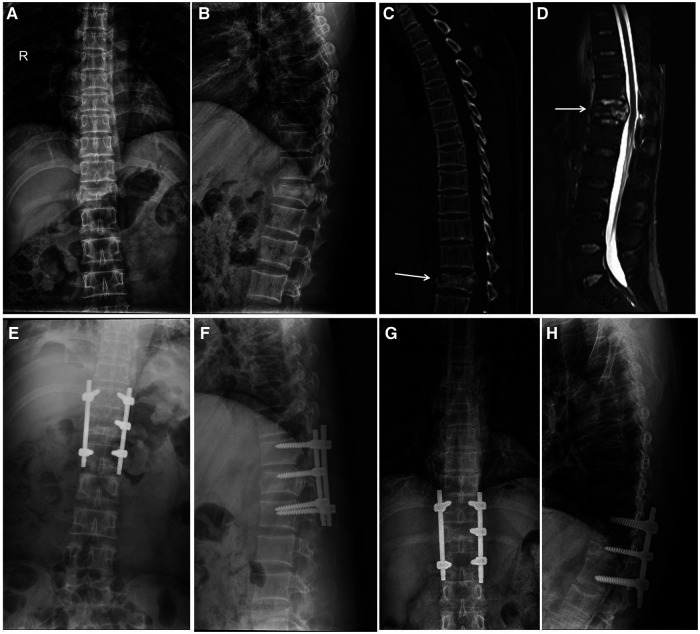
A 46-year-old male patient with T12 fracture and a decrease in anterior vertebral height >50% before surgery. (**A–D**) The preoperative x-ray, CT, and MRI images showed burst fracture of the T12 vertebra. (**E,F**) The patient underwent short-segment fixation including the fractured vertebra without transpedicular bone grafting. Immediate postoperative x-ray examination indicated that the height of T12 vertebral body was recovered. (**G,H**) x-ray examination, performed 2 year later, showed that the fracture vertebral body had collapsed and the height of the T12 vertebral body was decreased to 55.6%.

In this study, there were 67 patients in the unilateral fixation (5-screw fixation) group and 94 patients in the bilateral fixation (6-screw fixation) group. For patients with AO type A3/A4 fracture, no statistically significant differences were found between the unilateral and bilateral groups in terms of operative duration, intraoperative blood loss, preoperative and postoperative AVHR, VWA and LKA (*P *> 0.05). However, the AVHR of bilateral fixation group was greater than that of unilateral fixation group at the final follow-up (*P *< 0.001), and the LKA and VWA of bilateral fixation group were smaller compared with those of unilateral fixation group (*P *< 0.001, Table 1) (Figures 2, 3). These results meant that bilateral fixation can better maintain the height of fractured vertebra and the curve of the spine than those of unilateral fixation during the follow up.

**Table 1:** Comparison of the results between unilateral and bilateral fixation groups.

	Unilateral fixation group (*n* = 67)	Bilateral fixation group (*n* = 94)	*P*-value
Operative duration (min)	121.57 ± 83.32	136.91 ± 58.27	0.144
Intraoperative blood loss (ml)	210.43 ± 101.56	229.17 ± 96.77	0.237
AVHR (%)			
Preoperative (H1)	62.17 ± 13.67	61.89 ± 14.18	0.900
Postoperative (H2)	97.23 ± 4.89	98.76 ± 5.18	0.061
Final follow-up (H3)	**90.21 ** **± ** **14.19**	**96.25 ** **± ** **8.67**	**<0** **.** **001**
Correction loss (H2-H3)	**7.02 ** **± ** **11.03**	**2.51 ** **± ** **6.17**	**<0** **.** **001**
LKA (°)			
Preoperative (C1)	14.56 ± 7.18	14.47 ± 7.89	0.941
Postoperative (C2)	7.84 ± 4.62	6.56 ± 4.31	0.073
Final follow-up (C3)	**12.67 ** **± ** **8.57**	**8.43 ** **± ** **5.14**	**<0** **.** **001**
Correction loss (C3- C2)	**4.83 ** **± ** **5.78**	**1.87 ** **± ** **4.89**	**<0** **.** **001**
VWA (°)			
Preoperative (V1)	18.23 ± 5.58	18.74 ± 6.38	0.599
Postoperative (V2)	11.28 ± 4.02	10.15 ± 3.85	0.073
Final follow-up (V3)	**16.32 ** **± ** **7.15**	**12.39 ** **± ** **5.11**	**<0** **.** **001**
Correction loss (V3-V2)	**5.04 ** **± ** **3.17**	**2.24 ** **± ** **5.05**	**<0** **.** **001**

AVHR, the anterior vertebral height ratio of the fractured vertebra; LKA, local kyphotic cobb angle; VWA, vertebral wedge angle.

Bold values indicate the P values for the comparison between these two groups were <0.05.

### Different compression degrees of fractured vertebra

In order to determine the surgical outcomes of patients with different compression degree of fractured vertebra, we just included patients receiving bilateral fixation but no TBG for the comparison. There were 21 patients in group A (AVHC <30%), 25 patients in group B (AVHC ranged 30%–50%) and 22 patients in group C (AVHC >50%) (Table 2). The correction loss of AVHR, VWA and LKA at the last follow-up were significantly higher in group C than those recorded in the other two groups (*P *< 0.05) (Table 2), which indicated that patients with preoperative AVHC >50% of the injured vertebra had significant great correction loss during the follow up.

**Table 2:** Comparison of the results among different compression degrees of the fractured vertebra at final follow up (bilateral fixation).

Preoperative AVHC	*n*	Correction loss of the AVHR (%)	Correction loss of the LKA (°)	Correction loss of the VWA (°)
A (<30%)	21	1.31 ± 3.79	0.57 ± 3.16	1.03 ± 2.18
B (30%–50%)	25	2.14 ± 5.47	2.02 ± 4.18	2.65 ± 3.01
C (>50%)	22	9.39 ± 11.47*	5.82 ± 5.38*	5.87 ± 2.68*
*P*-value		0.031	0.045	0.028

AVHC, anterior vertebral height compressive ratio of the fractured vertebra; AVHR, the anterior vertebral height ratio of the fractured vertebra; LKA, local kyphotic cobb angle; VWA, vertebral wedge angle.

* *P* < 0.05 compared with group A and B.

### Transpedicular bone grafting for fractured vertebra

In the present study, there were 48 patients with AVHC >50% and received bilateral pedicle screw fixation. Among of these patients, 26 of them underwent TBG. There were no significant differences for the demographic data between patients with and without TBG, such as age, sex, and BMD. At the final follow up, the AVHR in patients with TBG was significantly higher than those without TBG (*P *= 0.003), but the correction loss of AVHR in TBG group was less than that of non-TBG group (*P *= 0.002). For the LKA and VWA, patients with TBG had smaller LKAs and VWAs compared with those without TBG (*P *< 0.05), and the correction loss in TBG group was less than those of non-TBG group (*P *< 0.05) at the final follow-up (Table 3).

**Table 3:** Comparison of the results between patients with and without TBG for those with AVHC >50%.

	SSPFI (*n* = 22)	SSPFI + TBG (*n* = 26)	*P*-value
AVHR (%)			
Preoperative (H1)	43.61 ± 8.32	44.68 ± 5.67	0.564
Postoperative (H2)	95.82 ± 5.14	98.44 ± 8.25	0.226
Final follow-up (H3)	**86.43 ** **± ** **10.26**	**95.18 ** **± ** **8.92**	**0.003**
Correction loss (H2-H3)	**9.39 ** **± ** **11.47**	**3.26 ** **± ** **4.51**	**0.002**
LKA (°)			
Preoperative (C1)	17.71 ± 6.81	16.52 ± 9.46	0.625
Postoperative (C2)	7.64 ± 4.82	5.47 ± 4.71	0.122
Final follow-up (C3)	**13.46 ** **± ** **7.51**	**6.52 ** **± ** **5.16**	**<0.001**
Correction loss (C3-C2)	**5.82 ** **± ** **5.38**	**1.05 ** **± ** **4.59**	**0.002**
VWA (°)			
Preoperative (V1)	23.12 ± 4.72	23.34 ± 6.15	0.892
Postoperative (V2)	12.32 ± 4.14	10.42 ± 3.18	0.079
Final follow-up (V3)	**18.19 ** **± ** **5.59**	**11.94 ** **± ** **4.31**	**<0.001**
Correction loss (V3-V2)	**5.87 ** **± ** **2.68**	**1.52 ** **± ** **5.04**	**0.001**

SSPFI, short-segment posterior fixation including the fractured vertebra; TBG, transpedicular bone grafting; AVHC, anterior vertebral height compressive ratio of the fractured vertebra; AVHR, anterior vertebral height ratio of the fractured vertebra; LKA, local kyphotic Cobb angle; VWA, vertebral wedge angle.

Bold values indicate the P values for the comparison between these two groups were <0.05.

## Discussion

Pedicle screw fixation is a safe and effective method for the surgical treatment of thoracolumbar fracture (16). Traditionally, thoracolumbar spine fractures are treated by long-segment posterior fixation (LSPF). However, LSPF results in lots of segments motor loss and is associated with a high risk of adjacent segment degeneration. In contrast, SSPF is characterized by a shorter operative duration and less amount of intraoperative blood loss. Also, it keeps more segments motor and exerts a similar effect compared with those treated with LSPF (9, 17). Aly et al. (18) conducted a meta-analysis to compare the clinical and radiological results of LSPF and SSPF. These results showed that there were no significant differences in imaging results, functional results, neurological status improvement and implantation failure rate between these two groups. Dobran et al. (19) compared the clinical effects of SSPFI and LSPF, and found that there was no significant difference in the correction of vertebral body height and Cobb angle between these two groups.

Traditional SSPF was conducted without placing pedicle screws in the fractured vertebra and was associated with a high rate of implant failure (5). SSPF including fractured vertebra (SSPFI) could increase the biomechanical stability of the fractured vertebral body and better maintains the stability of the spinal column, which decreases the incidence of postoperative implant failure (20). Kanna et al. (21) stated that the height of the injured vertebra was significantly recovered and the Cobb angle was well corrected after SSPFI. Ökten et al. (4) suggested that the improvement in kyphosis angle, sagittal index and compression ratio of the anterior vertebral height observed in the SSPFI group were superior to that recorded in the SSPF group. In the present study, the height of the anterior vertebra and the local kyphotic Cobb angle were significantly improved in all patients after the surgery, which were in line with previous studies.

However, are there any differences between the unilateral and bilateral pedicle screw placement in the fractured vertebra after the SSPFI surgery? Su et al. (22) used finite element models to investigate the biomechanics of unilateral short-segment fixation for stable thoracolumbar burst fracture, and found that short-segment fixation combined with intermediate unilateral fixation was a feasible treatment strategy for stable thoracolumbar fracture. Sun et al. (23) revealed that there was no significant difference in VAS score, Oswestry Disability Index, height of the injured vertebra and Cobb angle between the unilateral and bilateral pedicle screw fixation groups. But it was a small sample size study that just included 42 patients. In the present study, for patients with AO type A3/A4 fracture, the AVHR was greater, and the LKA and VWA were smaller in patients with bilateral fixation than those with unilateral fixation in the fractured vertebrae at the final follow-up. These results indicated that SSPFI using bilateral fixation in the fractured vertebra was superior to unilateral fixation in maintaining the height of the injured vertebra and the stability of the spine for the patient with AO type A3/A4 fracture. Therefore, we suggested that the pedicle screw should be placed on both sides as much as possible if the pedicles of the fractured vertebral body were intact.

Although SSPFI can effectively reduce the incidence of complications related to implant failure compared with SSPF (6, 19, 24), patients with a large preoperative compression degree of the fractured vertebra were more likely to lose the correction of the anterior height after the surgery. Kim et al. (25) studied the efficacy of SSPFI in the treatment of unstable thoracolumbar burst fractures and found that the recurrence rate of kyphosis significantly increased in patients with a Leukemia stem cell score >6 (26), suggesting that patients with collapse of the vertebral body in the sagittal plane >30% may have a higher risk of kyphosis recurrence. In the current study, we found that the correction loss of the AVHR, LKA and VWA were more obvious in patients with AVHC >50% at the final follow-up, which meant patients with AVHC >50% was not sufficient to restore the stability of the anterior spinal column even though bilateral fixation performed.

It remains controversial whether TBG can reduce the correction loss and implant failure in thoracolumbar fractures (12, 27–29). Advocates for TBG insist that it increases bone mass and effectively restore vertebral body height (11). Moreover, it reduces hardware failure and correction loss (28). However, opponents have reported opposite results, emphasizing that TBG did not decrease the loss of correction (30). In our study, for bilateral fixation patients with AVHC >50%, the correction loss of AVHR, VWA and LKA in patients with TBG were less than those without TBG at the final follow-up. Therefore, we draw a conclusion that TBG in fractured vertebra can decrease the correction loss of vertebral height for patients with a preoperative AVHC >50%. For serious vertebral compression or severe vertebral comminution cases, TBG was recommended to improve the outcomes.

The present study also has some limitations. Firstly, this study was conducted in a single institution while further research will benefit from a large sample size from multiple hospitals participating. Secondly, the follow-up period for the patients was not long enough (mean: 25.2 months). Hence, further investigation is warranted to assess the long-term effect of TBG on the treatment of single-level thoracolumbar fractures.

## Conclusion

SSPFI is an effective surgery for patients with single-level thoracolumbar fracture. Bilateral pedicle screw fixation is more effective than unilateral fixation in maintaining the vertebral height and the stability of the spinal column for AO type A3/A4 fracture. For patients with AVHC >50%, the correction loss of the vertebral height, local kyphotic Cobb angle and vertebral wedge angle at the final follow-up was more obvious, which can be reduced by TBG.

## Data Availability

The raw data supporting the conclusions of this article will be made available by the authors, without undue reservation.
